# Identification and characterization of alternative *STK39* transcripts within human and mouse kidneys reveals species‐specific regulation of blood pressure

**DOI:** 10.14814/phy2.14379

**Published:** 2020-02-28

**Authors:** Carlo J. Mercado, Xiaochun Wang, Paul R. Grimm, Paul A. Welling, Yen‐Pei C. Chang

**Affiliations:** ^1^ Division of Endocrinology, Diabetes and Nutrition University of Maryland School of Medicine Baltimore MD USA; ^2^ Departments of Physiology and Medicine Johns Hopkins University School of Medicine Baltimore MD USA

**Keywords:** *STK39*, transcriptional regulation

## Abstract

*STK39* encodes a serine threonine kinase, SPAK, which is part of a multi‐kinase network that determines renal Na^+^ reabsorption and blood pressure (BP) through regulation of sodium‐chloride co‐transporters in the kidney. Variants within *STK39* are associated with susceptibility to essential hypertension, and constitutively active SPAK mice are hypertensive and hyperkalemic, similar to familial hyperkalemic hyperkalemia in humans. SPAK null mice are hypotensive and mimic Gitelman syndrome, a rare monogenic salt wasting human disorder. Mice exhibit nephron segment‐specific expression of full length SPAK and N‐terminally truncated SPAK isoforms (SPAK2 and KS‐SPAK) with impaired kinase function. SPAK2 and KS‐SPAK function to inhibit phosphorylation of cation co‐transporters by full length SPAK. However, the existence of orthologous SPAK2 or KS‐SPAK within the human kidney, and the role of such SPAK isoforms in nephron segment‐specific regulation of Na^+^ reabsorption, still have not been determined. In this study, we examined both human and mouse kidney transcriptomes to uncover novel transcriptional regulation of *STK39*. We established that humans also express *STK39* transcript isoforms similar to those found in mice but differ in abundance and are transcribed from human‐specific promoters. In summary, *STK39* undergoes species‐specific transcriptional regulation, resulting in differentially expressed alternative transcripts that have implications for the design and testing of novel SPAK‐targeting antihypertensive medications.

## Graphical abstract

RNA‐seq analysis of human and mouse kidneys detects uncharacterized STK39 transcripts with species‐specific alternative first exons. Even though the resulting transcripts differ in 5'UTRs and transcriptabundance , they encoded similar proteins with truncated domains.

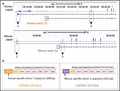

## 1 INTRODUCTION

Essential hypertension (EH) affects more than 25% of adults worldwide, and is responsible for an estimated 13.5 million deaths, equating to half of all cardiovascular deaths each year (Cabrera et al., 2015). Despite the wide variety of commonly used antihypertensive drugs, the prevalence of EH is steadily increasing due to ineffective, untailored treatments, and increased environmental risk factors (Shattuck, 2009). This pushes the need to better understand individual patient's unique mixture of genetic susceptibility and environmental factors, and to develop novel therapeutics that significantly improve the blood pressure (BP) control and decrease the risk of dangerous side effects. A previous genome wide‐association study conducted in the Old Order Amish identified strong associations between systolic BP and common variants in the gene, *STK39*, which encodes for a serine/threonine kinase known as STE20/SPS1‐related proline/alanine‐rich kinase (SPAK) (Wang, O'Connell, et al., 2009). The most likely functional variant demonstrated the increased transcriptional activity of *STK39* in vitro and is predicted to enhance *STK39* transcript (and SPAK protein) abundance (Cunnington et al., 2009; Wang, O'Connell, et al., 2009). Genetic activation of SPAK by a knock‐in mutation in mice causes hypertension and hyperkalemia, which recapitulates Familial Hyperkalemic Hypertension in humans (Grimm, Coleman, Delpire, & Welling, 2017). Furthermore, genetic inactivation or knockout of SPAK within mice results in lower BP (Geng, Hoke, & Delpire, 2009; Rafiqi et al., 2010), similar to Gitelman Syndrome in humans. These findings make SPAK a viable therapeutic target for novel antihypertensive medications.

SPAK and a related serine/threonine kinase, oxidative stress responsive 1 protein (OSR1) are components of the renal regulation of NaCl reabsorption. These kinases directly phosphorylate and activate the distal convoluted tubule (DCT)‐specific Na^+^‐Cl^‐^ co‐transporter (NCC) and the thick ascending limb (TAL)‐specific Na^+^‐K^+^‐2Cl^‐^ co‐transporter (NKCC2) (Delpire & Gagnon, 2008). Both NCC and NKCC2 reside at the apical membrane of renal epithelial cells, are direct targets of commonly used BP‐lowering diuretic drugs (thiazide‐ and loop‐diuretics, respectively), and are dysregulated in rare monogenic forms of hypotension (Gitelman‐ and Bartter syndromes, respectively) (Morla, Edwards, & Crambert, 2016; Simon, Karet, et al., 1996; Simon, Nelson‐Williams, et al., 1996). Previous studies in mice demonstrated how SPAK and OSR1 differentially regulate NCC and NKCC2, due to the presence of N‐terminally truncated SPAK isoforms known as SPAK2 and kidney‐specific (KS)‐SPAK (Grimm et al., 2012; McCormick et al., 2011; Saritas et al., 2013). While SPAK2 is thought to be alternatively translated from the mouse FL‐SPAK transcript, KS‐SPAK is derived from a mouse *STK39* transcript isoform that is transcribed from an alternative promoter within intron 5 (Grimm et al., 2012; McCormick et al., 2011). Both SPAK2 and KS‐SPAK lack varying amounts of the N‐terminal kinase domain and are predominantly expressed in the TAL, where they impede the binding of full length (FL)‐SPAK and OSR1 to NKCC2, thus inhibiting the NKCC2 phosphorylation and function (Grimm et al., 2012; McCormick et al., 2011). Conversely, FL‐SPAK is mainly expressed in the DCT and thus displays a greater interaction with NCC than with NKCC2, resulting in NCC activation (McCormick et al., 2011; Park, Curry, & McCormick, 2013; Saritas et al., 2013). Importantly, SPAK knock‐in mice that express inactive SPAK (SPAK^243A/243A^) exhibit decreased phosphorylation of both NKCC2 and NCC due to abolished FL‐SPAK kinase activity in both the DCT and TAL, but unaffected inhibition of OSR1 by SPAK2 and KS‐SPAK in the TAL (Rafiqi et al., 2010). In contrast, global knockout of SPAK completely abolishes the expression of FL‐SPAK and the inhibitory SPAK isoforms, resulting in decreased NCC phosphorylation, uninhibited OSR1 kinase activity in the TAL, and subsequent hyperphosphorylation of NKCC2 (Grimm et al., 2012; McCormick et al., 2011; Yang et al., 2010).

These findings highlight nephron segment‐specific regulation of NCC and NKCC2 by alternative SPAK isoforms and OSR1 in mice. However, it is not known if shorter orthologous SPAK isoforms exist in the human kidney, and if so, whether or not they play similar nephron segment‐specific functions as what was observed in mice. Smaller inhibitory SPAK products may also be generated from proteolytic cleavage (Markadieu et al., 2014), but whether or not these cleavage products physiologically regulate SPAK in human or mouse kidneys remains to be determined. Our limited understanding of the pretranslational and posttranslational regulation of human SPAK hinders the development of SPAK‐targeting therapeutic compounds. Here, we profiled the kidney transcriptomes of humans and mice in order to identify all uncharacterized transcript isoforms, including those derived from *STK39*. We used RNA‐seq, which does not rely on existing knowledge of transcripts, allowing for the detection of novel transcripts and splicing events, and validated novel transcripts with traditional methods such as RT‐PCR and 5’ rapid amplification of cDNA ends (RACE). Identification and characterization of both human and mouse *STK39* transcripts described here sheds light upon SPAK‐mediated BP control, providing further insights for the development of SPAK‐targeting antihypertensive medications.

## 2 MATERIALS AND METHODS

### 2.1 RNA isolation and quality control

Total RNA was purified from human and animal tissues using the RNAqueous^®^‐4PCR kit (Ambion) following the manufacturer's instructions. Following RNase I treatment, overall integrity of total RNA was determined based on RNA integrity number (RIN) using a Bioanalyzer RNA Nano Chip (Agilent) and only samples with a RIN >7 are used throughout this study.

### 2.2 Human and mouse kidneys used for RNA‐seq analysis

Eight RNA samples from different kidneys (four from humans and four from mice) were subjected to RNA‐seq analysis. Three of the four human RNAs were derived from cortices of postmortem kidney samples purchased from the National Disease Research Interchange (snap frozen 5–9 hr postmortem) and the fourth human kidney RNA was derived from a whole kidney surgical section from the University of Maryland Medical Center (with IRB approval). These kidney RNA samples had RINs from 7.3 to 8.6 (mean = 8). The four mouse kidney RNAs were derived from two sets of cortex and medulla samples from two different mice (male C57BL6, 8–10 weeks old), and all had RINs from 9.1 to 10 (mean = 9.7). All procedures involving mice were approved by the University of Maryland School of Medicine Institutional Animal Care and Use Committee (IACUC).

### 2.3 RNA‐seq analysis

Total RNA (2 µg) from each human and mouse kidney was reverse transcribed into cDNA with an oligo‐dT primer to capture poly(A) transcripts. cDNA was then fragmented, adapter‐ligated, and organized into cDNA libraries. To achieve the desired sequence depth necessary for low‐abundance transcript and novel transcript isoform detection (>80 million mapped reads per sample) (Sims, Sudbery, Ilott, Heger, & Ponting, 2014; Trapnell et al., 2010), four samples were sequenced and pooled in a single lane of flow cell. Tagging of each fragment library with a unique 6 base‐pair (bp) code allowed for multiplexed high‐throughput sequencing of cDNA using the Illumina HiSeq^®^ 2500, in which 100 bp from both ends of each fragment were sequenced. Analysis of sequence reads was conducted using the transcriptome analysis pipeline of the Institute of Genome Sciences (IGS). TopHat (version 1.4.0) and Bowtie (version 0.12.9) (Trapnell et al., 2010) software packages aligned sequence reads to the reference genome (either the GRCh37 human genome assembly or the GRCm38 mouse genome assembly), and also generated contigs from the reads to establish the transcript structure (i.e.: exon‐exon junctions) (Wang, Gerstein, & Snyder, 2009). Transcript abundance was calculated using Cuffllinks (version 1.3.0) software, and quantification scores for all genes and transcript isoforms were presented as fragments per kilobase of exon per million mapped reads (FPKM) values. To allow for the detection of novel and alternatively spliced transcripts, assemblies and abundance estimations were performed without existing gene annotation data. Cuffcompare and Scripture software packages were utilized to compare the putative novel transcripts to known annotated transcripts. The expression profiles for all known and novel transcripts detected in human and mouse kidneys were visualized using the Integrative Genomics Viewer (IGV) (Anders & Huber, 2010). Both human and mouse kidney transcriptome datasets from our RNA‐seq analysis have been deposited into the National Center for Biotechnology Information's Gene Expression Omnibus (GEO) database (series accession number http://www.ncbi.nlm.nih.gov/geo/query/acc.cgi?acc=GSE106548 for both human and mouse datasets) and are available to the scientific community.

### 2.4 Human tissues used for validation and characterization studies

Validation and characterization studies of human kidney expressed SPAK mRNA and protein isoforms were performed using “medically wasted” human kidneys. The Living Legacy Foundation (LLF) coordinates organ donations in which all transplantable organs are recovered from donors who have consented to multiple organ donations. Occasionally, organs originally recovered for transplantation may not be suitable for transplantation and are considered “medically wasted,” but were pre‐authorized for use in research. So far, we obtained six kidneys from four LLF donors (averaged RIN >8), which provided total RNA and protein for downstream RT‐PCR and western blot analyses. In addition, total RNA was also isolated from human heart, subcutaneous fat, whole blood, and brain (frontal cortex). Commercially available multi‐tissue cDNA or mRNA panels were not used in this study due to their suboptimal quality compared to RNAs isolated from human tissues we obtained. Human heart and subcutaneous fat RNAs were provided by the Mid‐Atlantic Nutrition Obesity Research Center of the University of Maryland School of Medicine, and the human frontal cortex tissue samples were provided by Dr. Todd Gould and the developmental brain bank of the University of Maryland, Baltimore. Human whole blood samples were obtained from healthy members of the Old Order Amish community.

### 2.5 Phenotypic information of human kidneys

Both phenotypic information and medical history of all human kidney donors used for RNA‐seq and validation are summarized in Table S1. The mean age of all human donors (four males and four females) was 56 years of age. Four out of eight human kidneys were from individuals with a history of hypertension. Kidneys from African Americans as well as European Americans were included in our study. The surgical section came from the noncancerous part of a kidney removed due to renal cell carcinoma. Otherwise, none of the kidneys in our study had any known kidney diseases.

### 2.6 Nonhuman primate kidneys

Kidneys from two rhesus monkeys (6‐year‐old female and 7‐year‐old male) were obtained from Dr. Agnes Azimzadeh's laboratory. As part of Dr. Azimzadeh's study, both monkeys received a single dose of a monoclonal antibody that depleted B (CD20+) cells 90 days before euthanasia. At the time of euthanasia, both monkeys were of normal healthy weight (5.9 kg and 12.4 kg, respectively), with normal kidney function. The use of nonhuman primate tissue was approved by the IACUC of the University of Maryland School of Medicine.

### 2.7 5’ rapid amplification of cDNA ends (RACE) of human kidney RNA

In addition to RNA‐seq, 5’RACE was used to validate the expression of alternative 5’ ends of candidate human *STK39* transcript isoforms. 5’RACE of human kidney RNA, including cDNA synthesis, amplification, and characterization of 5’RACE products through molecular cloning, were conducted using the FirstChoice^®^ RLM‐RACE kit (Ambion) based on manufacturer's instructions. 5’RACE‐amplified products were visualized through agarose gel electrophoresis. PCR products from novel 5’ ends of transcripts were gel purified and cloned into the pCR^TM^2.1‐TOPO^®^ (Invitrogen) cloning vector and sequenced using gene‐specific primers or primers off the cloning vector.

### 2.8 Reverse transcriptase (RT)‐PCR validation of uncharacterized STK39 transcripts

Total RNA (≥1 µg) was reverse transcribed into cDNA using the Transcriptor First Strand cDNA Synthesis kit (Roche) following the manufacturer's instructions. Forward and reverse primers targeted either all transcripts for a specific gene, or isoform‐specific sequences derived from alternative promoter usage or splicing (Table S2). PCR was conducted using the standard protocol provided for GoTaq^®^ Green Master Mix (Promega). RT‐PCR amplified products were visualized through agarose gel electrophoresis.

### 2.9 Quantitative (q)RT‐PCR

Quantitative (q)RT‐PCR was conducted using the LightCycler^®^ 480 SYBR Green I Master protocol (Roche). Quantification of alternative *STK39* transcripts using isoform‐specific primers was normalized to the quantification of total *STK39* mRNA within humans or mice, or to the quantification of a housekeeping gene such as β‐actin or GAPDH (TaqMan probes obtained from Applied Biosystems). TaqMan assays were conducted using the LightCycler^®^ 480 Probes Master protocol (Roche). qRT‐PCR was performed in triplicate wells per sample. Relative abundance of transcript isoforms was determined as the average of triplicate qRT‐PCR results (2^−∆∆CT^) ± standard deviation (*SD*) for each sample.

### 2.10 Cell culture and transfection

HEK293A, Hela, and Huh7 cells (American Type Culture Collection) were cultured in complete medium, consisting of 4.5 g/L glucose, L‐glutamine, and sodium pyruvate Dulbecco's Modified Eagle Medium (DMEM, Cellgro) supplemented with 10% fetal bovine serum (Gibco). Cells were seeded at 1.5 × 10^5^ cells/well in 24‐well plates and were maintained at 37ºC and 5% CO_2_. Transfections of plasmids within each cell line were performed using the Lipofectamine 2000 transfection reagent (Invitrogen) following the manufacturer's instructions.

### 2.11 Luciferase reporter gene assay

The alternative promoter regions within human *STK39* based on newly discovered transcription start sites (TSSs) and in silico analysis were experimentally validated using a luciferase reporter gene vector (pGL4.10, Promega). Candidate promoter sequences were PCR amplified from human genomic DNA using forward and reverse primers that were tagged with 16 bp 5’‐overhangs identical to a double digested region of pGL4.10 (XhoI/HindIII) (Table S3). This facilitated the directional cloning of each gel purified promoter fragment upstream of a firefly luciferase cassette by the In‐Fusion Cloning System (Clontech). Sequences and orientations of all promoter inserts were confirmed through Sanger sequencing.

Each luciferase reporter construct (800 ng) was individually co‐transfected (in triplicate) with 8 ng of the pRL‐SV40 *Renilla* luciferase reporter vector (Promega) into HEK293A cells. After 48 hr post‐transfection, cells were lysed and processed using the Dual Luciferase^®^ Reporter Assay System (Promega) following manufacturer's instructions. Firefly and *Renilla* luciferase activities were measured, in triplicate, using a VICTOR^TM^ X3 Multilabel Plate Reader (Perkin Elmer 2030). Firefly luciferase activity was normalized to *Renilla* luciferase activity to control for experimental variation in transfection efficiencies and data from three independent transfections were normalized to that of empty vector transfected cells (set to 1). Reporter constructs that were positive for promoter activity within HEK293A cells were also assayed in Hela and Huh7 to assess differential promoter activity in different cellular environments.

### 2.12 Western blot analysis

Human and animal kidney tissues were homogenized in RIPA protein lysis buffer (Teknova), supplemented with 1X Halt Protease and Phosphatase Inhibitor Cocktail. Cell lysate protein concentration was determined using the Pierce^TM^ BCA Protein Assay Kit (ThermoFisher Scientific). Protein lysates were solubilized in either 5X Lane Marker Reducing Sample Buffer (ThermoFisher Scientific) or in 2X Laemmli Sample Buffer (BioRad). Solubilized proteins (20–25 µg of crude lysate) were resolved by size on SDS‐PAGE, 10% Tris‐HCl protein gels (BioRad), and transferred onto polyvinylidene difluoride (PVDF) membranes (Fisher Scientific). After blocking in milk, membranes were hybridized with an anti‐SPAK antibody that specifically targets the C‐terminus (Anders & Huber, 2010), which was used against human and animal kidneys to compare the SPAK protein expression profiles of different species. Visualization of SPAK protein isoforms was conducted using the FluorChem^TM^ Q System (Alpha Innotech). Bands were assigned to putative N‐terminally truncated SPAK isoforms based on their predicted sizes. Densitometry analysis was performed for each band using the FluorChem^TM^ Q SA software (Alpha Innotech), and band intensity was normalized relative to an internal control, such as α‐tubulin or β‐actin (Sigma‐Aldrich).

### 2.13 Statistical analysis

Statistical significances of differences in promoter activity, transcript abundance, and protein abundance were determined by the Student's unpaired two‐tailed *t* test, with *p* values of <.05 considered significant.

## 3 RESULTS

### 3.1 RNA‐seq alignment, quality control, and gene expression characteristics

Both human and mouse samples generated a robust averaged sequencing depth of >90 million mapped read‐pairs, and an averaged percentage of both mapped and exonic reads of >85% (Figure S1a,b). These characteristics are expected based on study design and indicate high‐quality RNA‐seq datasets (Piechotta, Lu, & Delpire, 2002; Sultan et al., 2008). While a small percentage of reads mapped to intronic regions (average of ~7.2% and ~4.1% for humans and mice, respectively), this was expected and typical of other published RNA‐seq results, since preparations of mature mRNA may include partially processed nuclear RNAs, and some genes may have internal exons that have yet to be annotated in gene models (Thiagarajan et al., 2011).

Sample cluster analysis of the RNA‐seq data revealed that human Sample 4 (whole kidney) clustered separately from the human cortex samples (Human Samples 1–3), as expected. However, among the cortex samples, Human Sample 1 clustered separately from Human Samples 2 and 3 (Figure S2a) most likely because Human Sample 1 is female derived, while Human Samples 2 and 3 are both male derived. In fact, the most differentially expressed gene between the three cortex samples and the one whole kidney sample (male derived) is *XIST*, which dictates X inactivation within mammalian females. In addition, the mouse samples demonstrated the expected clustering of replicates, in which Mouse Samples 1 and 3 (cortex) clustered separately from Mouse Samples 2 and 4 (medulla) (Figure S2a).

The distribution of gene expression levels was similar across all replicates of human and mouse samples (Figure S2b) and is consistent with prior RNA‐seq studies (Hart, Komori, LaMere, Podshivalova, & Salomon, 2013). Human samples had an average of ~18,000 transcripts with FPKM >1, while mouse samples had an average of ~15,000 transcripts, with FPKM >1.

### 3.2 Detection of uncharacterized STK39 transcripts in human and mouse kidneys through RNA‐seq and 5’RACE

**Figure 1:**
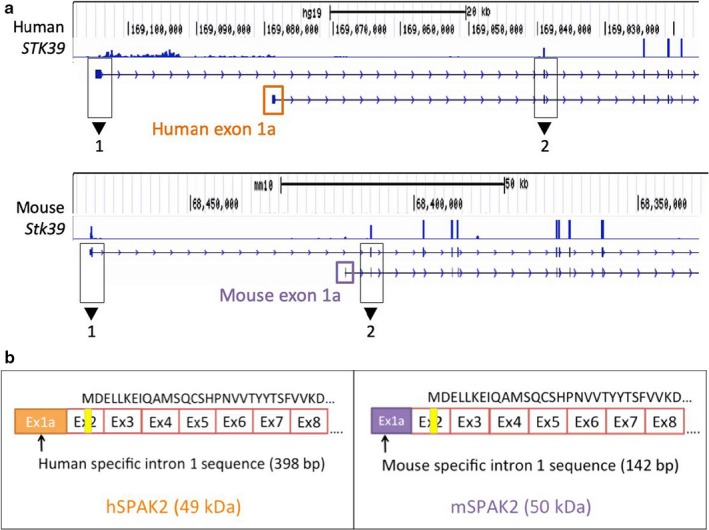
RNA‐seq analysis of human and mouse kidneys detects uncharacterized *STK39* transcripts with species‐specific alternative first exons. (a) For both human and mouse transcripts, sequence read depth is displayed. The x‐axis represents chromosomal location and the y‐axis represents overall depth of coverage. Peaks within the plots indicate high abundance of reads mapping to that location. Gene tracks for both the reference *STK39* transcript and the uncharacterized transcript are displayed directly underneath the coverage plots. Black boxes demarcate exons 1 and 2 in each transcript. Orange and purple boxes define alternative first exons for humans and mice, respectively. Coverage plots and gene tracks were obtained using the Integrative Genomics Viewer (IGV) and the UCSC Genome Browser. Data from Human Sample 1 and Mouse Sample 1 are shown. (b) Comparison between human and mouse uncharacterized *STK39* transcripts, which potentially encode for orthologous SPAK2 proteins. Exon 1a differs in sequence and in length between humans and mice. Vertical yellow lines indicate locations of putative alternative translation start sites. Protein sequences of the N terminus are displayed on top of each transcript schematic

Our RNA‐seq analysis revealed uncharacterized *STK39* transcript isoforms in both human and mouse kidneys. These include *STK39* transcripts with species‐specific alternative first exons (exon 1a) that potentially encode for SPAK2 proteins in both humans and mice (Figure 1a). Human exon 1a is derived from a 398 nt internal segment within intron 1 (~39.6 kb upstream of exon 2 in the genomic sequence). While mouse exon 1a is located within intron 1, it is only 142 nt in size, ~5.6 kb upstream of exon 2, and shares very limited sequence homology with the human exon 1a (26.8%).

Both human and mouse alternative *STK39* transcripts containing exon 1a are predicted to use the same alternative translation start sites in exon 2 and result in an N‐terminally truncated SPAK protein that is near identical in peptide sequence and size to the mouse SPAK2 isoform already described (McCormick et al., 2011; Simon, Nelson‐Williams, et al., 1996) (Figure 1b). While mouse SPAK2 is initially thought to be alternatively translated from the full‐length mouse *STK39* transcript (Piechotta, Garbarini, England, & Delpire, 2003), our data suggests that alternative transcripts with species‐specific TSSs encode for SPAK2 in both humans and mice. Therefore, we designated these transcripts as hSPAK2 and mSPAK2 for human and mouse SPAK2, respectively.

**Figure 2:**
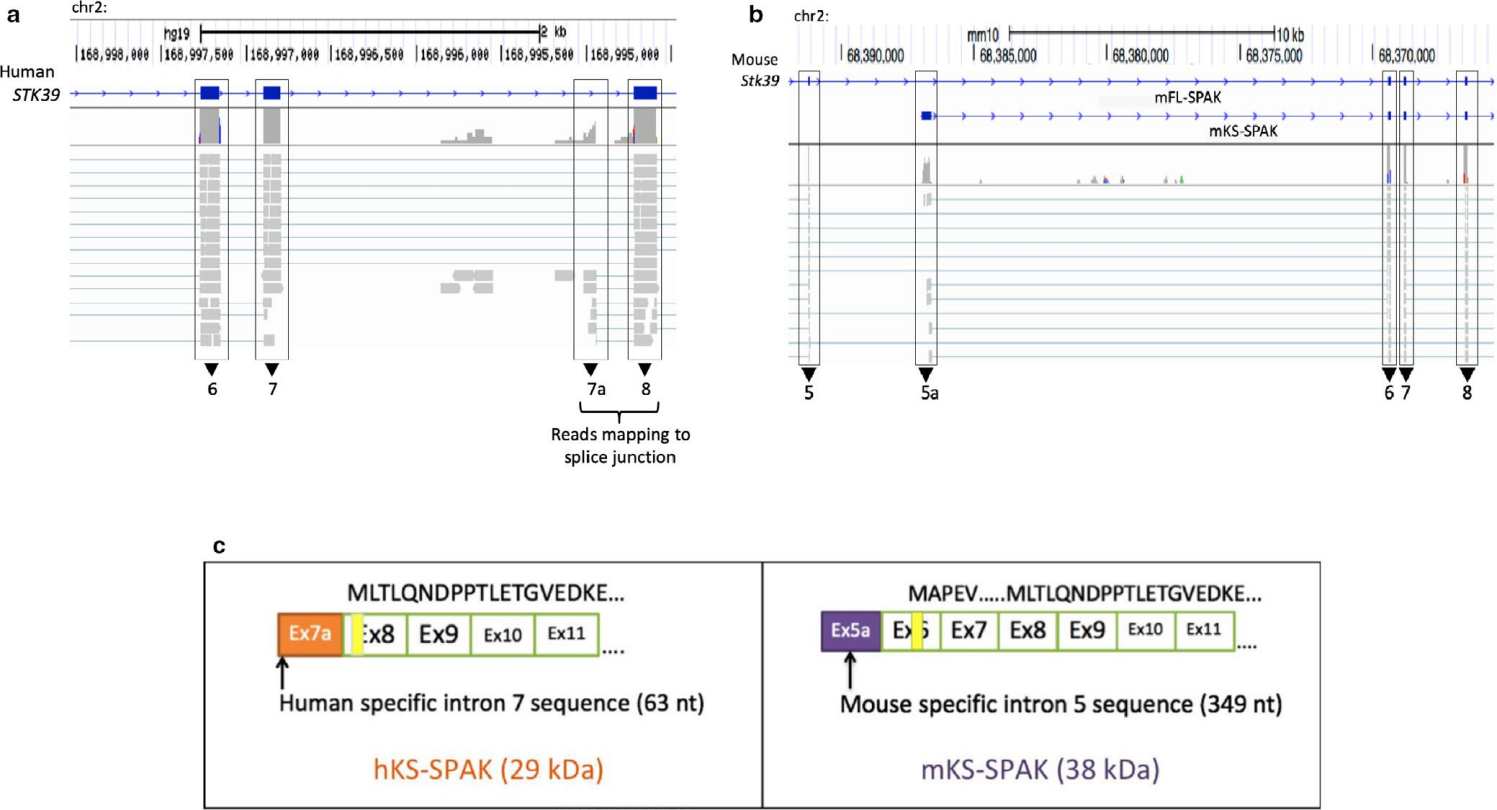
RNA‐seq analysis of human kidneys detects an uncharacterized 5’ truncated *STK39* transcript that resembles mouse KS‐SPAK. (a and b) display representative snapshots of IGV outputs for Human Sample 1 and Mouse Sample 1, respectively. Mapped reads and coverage surrounding exons (black boxes) are shown. (a) Sequence reads map to an uncharacterized splice junction in human *STK39* between an internal segment of intron 7 and the 5’ end of exon 8. (b) Sequence reads map to the previously characterized splice junction of exon 5a in mouse KS‐SPAK mRNA. (c) The predicted protein encoded by the shorter *STK39* transcript in humans is smaller than the protein encoded by the mouse KS‐SPAK transcript (263 amino acids vs. 306 amino acids, respectively). Protein sequences are displayed on top of each transcript schematic

Although a human homolog for KS‐SPAK did not appear in the final transcript assembly of the RNA‐seq data, 5’RACE of human *STK39* confirmed the presence of another transcription start site within intron 7, beginning with a novel exon (63 nt long), which we termed exon 7a. This exon is shorter and not homologous to exon 5a within mouse *Stk39*. RNA‐seq raw read alignments of human *STK39* revealed splice junctions with multiple reads spanning from exon 7a to exon 8. Similarly, junction reads were also observed that span mouse exons 5a and exon 6 (Figure 2a,b). While this novel human SPAK transcript isoform is transcribed from a nonhomologous exon, this 5’ truncated human *STK39* transcript potentially encodes for a protein that lacks almost the entire kinase domain and resembles mouse KS‐SPAK. Therefore, we refer this isoform as hKS‐SPAK (Figure 2c).

### 3.3 Alternative STK39 transcripts are differentially expressed across human tissues

**Figure 3:**
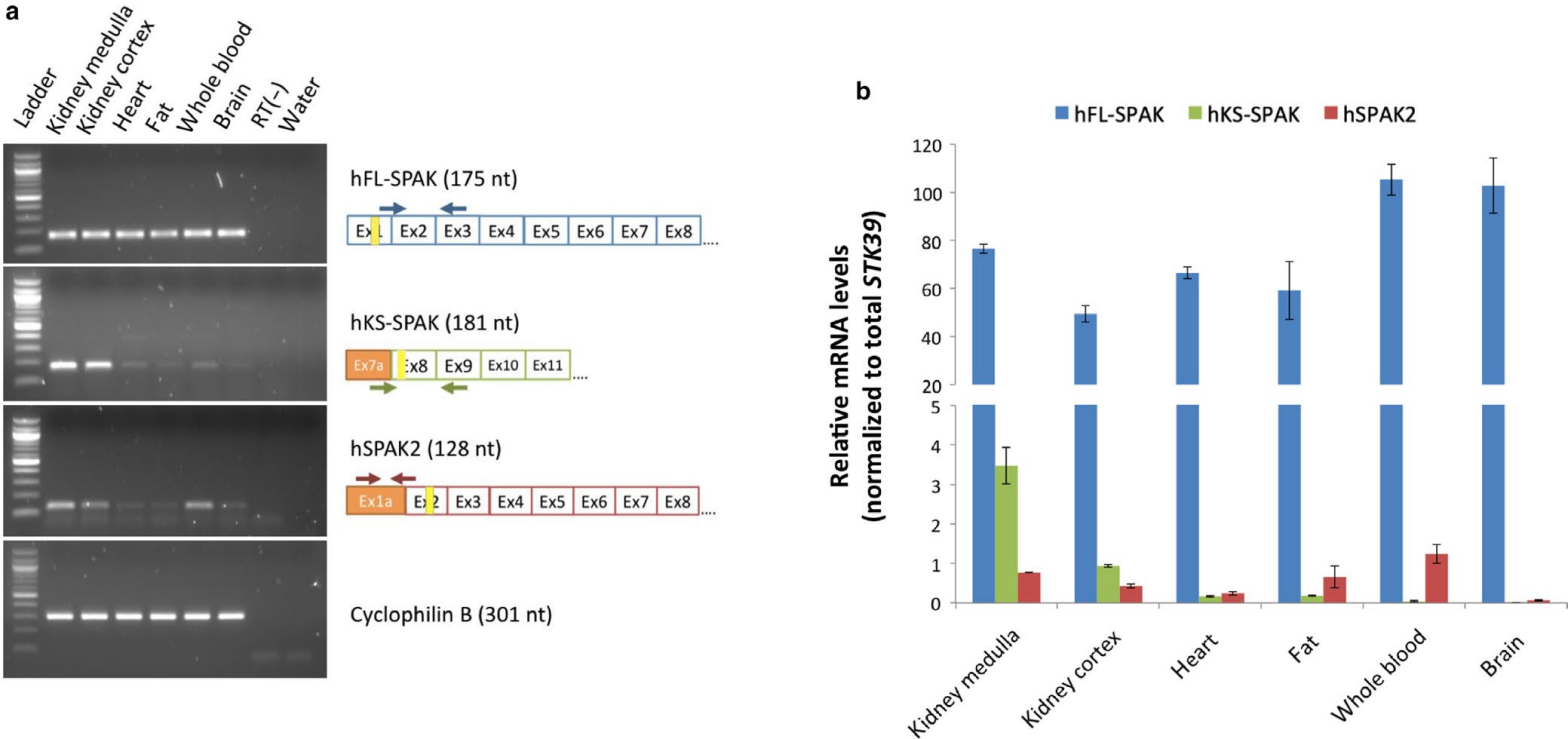
PCR amplification of alternative *STK39* transcripts reveals differential expression across human tissues. Transcript isoform‐specific forward and reverse primers were used for the amplification of human *STK39* transcript isoforms with alternative TSSs in both RT‐PCR (a) and qRT‐PCR (b) amplification. (a) Amplification of a housekeeping gene (cyclophilin B) in all human tissues is displayed below. In agarose gel images: Ladder is 100 bp DNA ladder (New England Biolabs); RT(‐) refers to no RT control for brain sample. In RT‐PCR schematics: Arrows represent forward and reverse primers; vertical yellow lines indicate locations of putative alternative translation start sites. (b) Similar mRNA expression levels for each human *STK39* isoform were observed within two biological replicates of each human tissue. Figure displays representative distribution in one set of human tissues. Results are given as the mean ± *SD* from triplicate qRT‐PCR results. All primer sets demonstrated comparable amplification efficiencies

The relative expression of *STK39* transcript isoforms was determined in human kidney (cortex and medulla), brain, subcutaneous fat, and whole blood using isoform‐specific primers. Conventional and qRT‐PCR revealed differential expression of human *STK39* transcript isoforms (Figure 3a,b). Compared to the full‐length transcript (hFL‐SPAK), hSPAK2 mRNA was detectable at a low level across all six tissues examined. Notably, hKS‐SPAK mRNA demonstrated robust expression only within human kidney, with very low expression seen in other tissues. Furthermore, quantification of hKS‐SPAK mRNA expression within two sets of human medulla and cortex samples revealed that the medulla expresses hKS‐SPAK mRNA at much higher levels compared to the cortex (Figure S3).

### 3.4 hFL‐SPAK and hKS‐SPAK TSSs demonstrate promoter activity in an in vitro reporter gene assay

**Figure 4:**
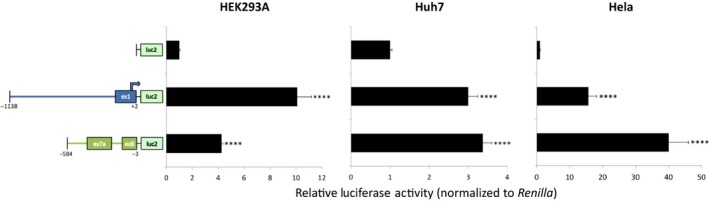
In promoter construct schematics: Numbers indicate nucleotide locations within genomic DNA, relative to the translation start codon within exon 1 of the hFL‐SPAK transcript and exon 8 of the hKS‐SPAK transcript; arrow indicates location of alternative translation start codon; *luc2* represents luciferase reporter gene cassette for pGL4.10. Results are given as the mean ± *SD* from 3 independent experiments. empty vector, ****p < .001 versus empty vector

For each human alternative *STK39* transcript, candidate promoter regions were selected based on in silico predicted regulatory profiles and tested using an in vitro reporter gene assay. As expected, the hFL‐SPAK promoter region demonstrated a significant increase in promoter activity over empty vector in three human cell lines (HEK293A, Huh7, and Hela) (Figure 4). Importantly, the promoter region of the hKS‐SPAK also demonstrated significant activity above the empty vector control. Promoter activity of the hSPAK2 transcript was not determined because of either spontaneous mutations or spurious translation start sites that occurred in all seven attempts to clone its promoter region, leading to disruption of luciferase translation, and lower luciferase activity than the empty vector control.

### 3.5 The human kidney expresses SPAK products that are consistent with N‐terminally truncated protein isoforms, hSPAK2, and hKS‐SPAK

**Figure 5:**
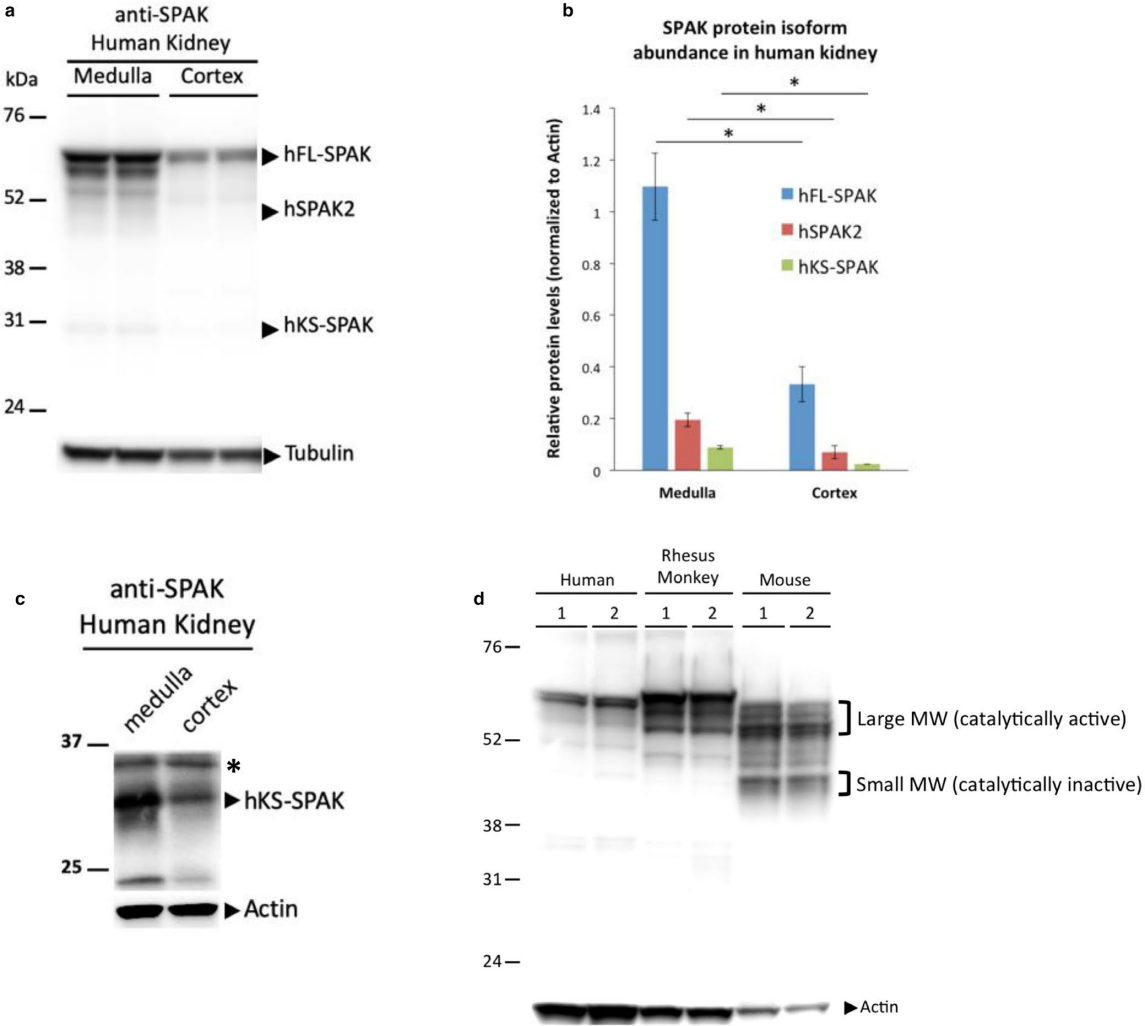
SPAK protein expression in the human kidney. (a) Representative western blot analysis using an anti‐SPAK antibody. (b) Quantification of human SPAK protein isoforms in medulla and cortex. Results are given as the mean ± *SD* from two biological replicates of each sample. *p < .05. (c) Representative western blot of a smaller sized protein. For this experiment, a different western blot from Figure A was cut below 37 kDa and hybridized with the same anti‐SPAK antibody. Besides hKS‐SPAK, a non‐specific band from either protein cleavage or cross‐reactivity is also detected and is marked with an asterisk*. (d) The same anti‐SPAK antibody was used against kidney lysates from humans, rhesus monkeys, and mice for a representative comparison of total SPAK protein expression in multiple species. Two biological replicates (1 and 2) of each species are shown. The same amount of protein lysate was loaded in each lane. Band intensities for actin reflect the sequence similarities between humans, rhesus monkeys, and mice. MW, molecular weight

To determine whether putative SPAK protein isoforms encoded by hSPAK2 and hKS‐SPAK are expressed in the human kidney, we used an anti‐SPAK antibody that recognizes the common C‐terminal region of SPAK (Piechotta et al., 2002). This allowed for the detection of all human SPAK protein isoforms with alternative N‐termini, including those encoded by *STK39* mRNA isoforms transcribed from alternative promoters, as well as the previously identified C‐terminal SPAK cleavage products (Markadieu et al., 2014). The C‐terminal antibody detected several bands that were consistent in size with alternative human SPAK isoforms, including the full‐length protein (hFL‐SPAK, ~59 kDa) and the putative N‐terminally truncated isoforms, hSPAK2 (~49 kDa), and hKS‐SPAK (~29 kDa) (Figure 5a). All three protein isoforms exhibited differential expression that resembled relative *STK39* mRNA levels in the human kidney, where hFL‐SPAK was the most abundant isoform at both the mRNA and protein levels (Figures 3b and 5b). Furthermore, the hKS‐SPAK protein was preferentially expressed in human medulla compared to human cortex (Figure 5c), which corresponded with hKS‐SPAK mRNA expression in all the kidney samples examined (Figure S3 and Figure 5b). When focusing on smaller proteins, an additional protein product <37 kDa was detected (Figure 5c, marked with an asterisk *). As a 36 kDa cleavage product has been previously reported (Markadieu et al., 2014), this band can either be a cleavage product or from cross reactivity to an unrelated protein.

### 3.6 Comparison of SPAK protein expression among different mammalian kidneys reveals species‐specific expression profiles, suggesting divergent evolution of SPAK

To examine the structural similarity of SPAK expression throughout different mammalian species, we analyzed kidney cortex lysates from humans, rhesus monkeys, and mice on the same western blot, in which the same C‐terminal SPAK antibody was used (Figure 5d). Consistent with previous reports of SPAK protein expression in mice (McCormick et al., 2011; Simon, Nelson‐Williams, et al., 1996), we observed two prominent clusters of SPAK isoforms in mouse kidneys, including a cluster of large molecular weight isoforms running between 55 and 60 kDa, and smaller molecular weight isoforms closer to 45 kDa. The assignment of specific protein bands to specific mouse SPAK isoforms varies from study to study, but the general consensus remains that the larger molecular weight isoforms correspond to catalytically active SPAK, while the smaller molecular weight isoforms correspond to catalytically inactive SPAK in mice (Figure 5d) (Markadieu et al., 2014; McCormick et al., 2011; Simon, Nelson‐Williams, et al., 1996; Wade et al., 2015).

Notably, while the SPAK protein expression profiles differed between human and mouse kidneys, rhesus monkeys shared certain similarities with both humans and mice with regards to SPAK expression in the kidney (Figure 5d). Human and rhesus monkey kidneys both expressed a SPAK isoform that most closely resembled FL‐SPAK at ~60 kDa, which was larger than any of the isoforms observed in mice. Rhesus monkeys expressed additional SPAK isoforms just below 60 kDa that were not as prominent in humans and were similar in size to the cluster of large molecular weight SPAK isoforms in mice. However, the cluster of small molecular weight isoforms (~45 kDa) in mice was not as prominent in either human or rhesus monkey kidneys. Taken together, these results demonstrated that humans, rhesus monkeys, and mice all express multiple SPAK isoforms in the kidney, which points to an evolutionarily conserved mechanism in the transcriptional regulation of *STK39*. However, since primates and rodents diverged, humans and mice acquired species‐specific TSSs for SPAK2 and KS‐SPAK, as well as species‐specific transcriptional regulation that lead to starkly different SPAK profiles observed in the kidneys for these different species.

## 4 DISCUSSION

Transcriptional regulation of the mouse *Stk39* gene results in alternative SPAK proteins with differing functions (Grimm et al., 2012; McCormick et al., 2011; Simon, Nelson‐Williams, et al., 1996). While this system has been well established within mouse models, the data presented here provide the first evidence of transcriptional regulation of this gene in humans. Our data demonstrated that alternative transcripts may encode for two kinase‐deficient proteins that are orthologous to the mSPAK2 and mKS‐SPAK proteins in mice. Human *STK39* transcript isoforms are differentially expressed across multiple human tissues, with the hKS‐SPAK transcript exhibiting the same preferential expression in kidney medulla that was observed with the mKS‐SPAK ortholog. In addition, the novel hKS‐SPAK promoter demonstrated in vitro transcription in all three cell lines tested.

Comparative analysis of SPAK protein expression among different mammalian kidneys demonstrated that humans and rhesus monkeys share very similar SPAK expression profiles, where smaller molecular weight SPAK isoforms are significantly less expressed than the larger molecular weight SPAK isoforms in both species. Conversely, smaller molecular weight SPAK isoforms in mouse kidneys were more abundantly expressed compared to human and rhesus monkey kidneys. These observations suggested that hSPAK2 and hKS‐SPAK proteins might play a different role in regulating BP than their mouse orthologs.

One possible compensatory mechanism for low expression of human KS‐SPAK in mice versus humans is that there is high expression of a 5’ truncated OSR1 that also lacks kinase activity. In fact, a short form of mouse OSR1 (S‐OSR1) was identified in the mouse kidney (Ferdaus et al., 2016). Our data did not reveal any evidence of this transcript in the human kidney. Mouse S‐OSR1 transcript starts with a novel exon 4a. There is no evidence of a homologous exon 4a in our four RNA‐seq data sets designed to identify low expressing or novel transcripts. While we cannot formally exclude the possibility that an inhibitory form of human S‐OSR1 exists, it is unlikely that such an isoform is expressed in a manner similar to mouse KS‐OSR1.

While smaller molecular weight SPAK isoforms are expressed in the human kidney, either through alternative *STK39* transcripts or previously described cleavage products (Markadieu et al., 2014), it is still unclear how hSPAK2 and hKS‐SPAK expression may be physiologically regulated to maintain the renal salt balance. These observations have major implications for the design and testing of novel SPAK‐targeting antihypertensive medications. SPAK represents an attractive pharmacological target for hypertensive patients, since it was previously demonstrated that genetically inactivating or knocking out SPAK in mice leads to lower BP, but relatively few side effects (Grimm et al., 2017; Rafiqi et al., 2010). Indeed, researchers have already begun testing the efficacy of SPAK targeting compounds within mouse models (Kikuchi et al., 2015). However, since humans and mice demonstrated species‐specific regulation of SPAK, it is possible that SPAK‐targeting compounds may produce unexpected results between both species. As such, researchers and pharmaceutical companies must take this into account, and consider using other model organisms, such as nonhuman primates or genetically “humanized” mouse models, that have similar SPAK‐expression profiles as humans when testing the therapeutic potential of SPAK‐targeting compounds in the future.
