# Supplementary material for: Identification and characterization of alternative STK39 transcripts within human and mouse kidneys reveals species‐specific regulation of blood pressure
Source: Physiol Rep. 2020 Feb 28;8(4):e14379. doi: 10.14814/phy2.14379 (PMC7048380; doi:10.14814/phy2.14379)
Supplement: Supplementary file 1 [file PHY2-8-e14379-s001.pdf]

S1A

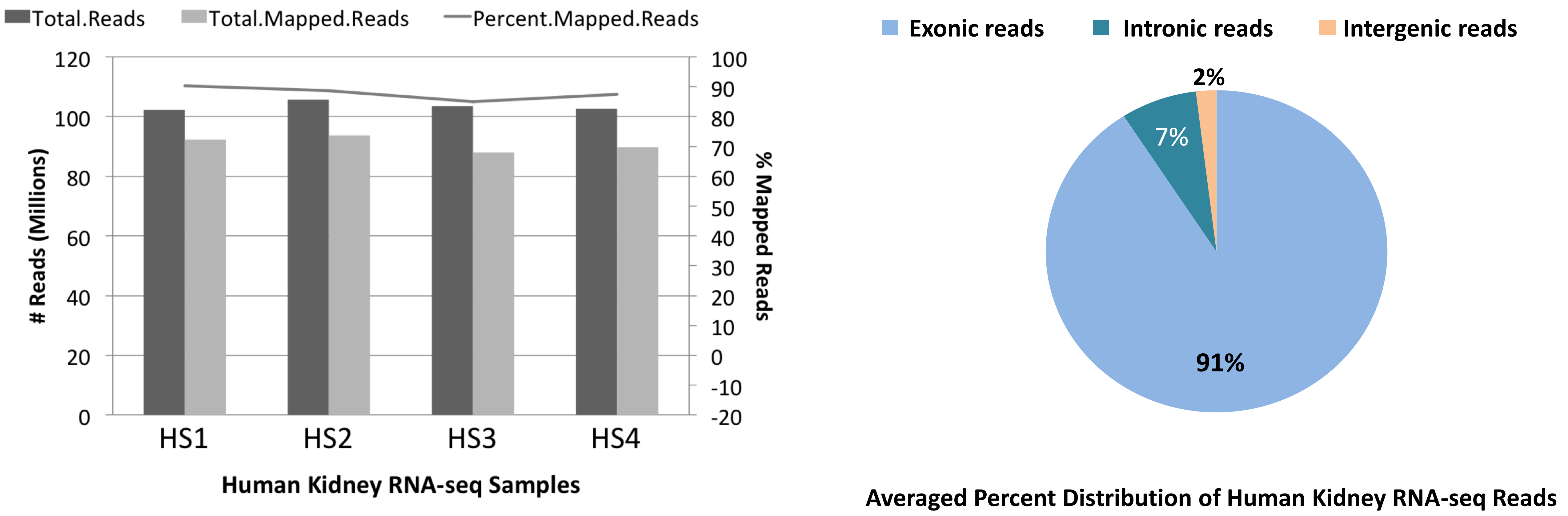

Total.Reads
Total.Mapped.Reads
Percent.Mapped.Reads
Reads (Millions)
% Mapped Reads
120
100
80
60
40
20
0
100
90
80
70
60
50
40
30
20
10
0
-10
-20
HS1
HS2
HS3
HS4
Human Kidney RNA-seq Samples
Exonic reads
Intronic reads
Intergenic reads
2%
7%
91%
Averaged Percent Distribution of Human Kidney RNA-seq Reads

S1B

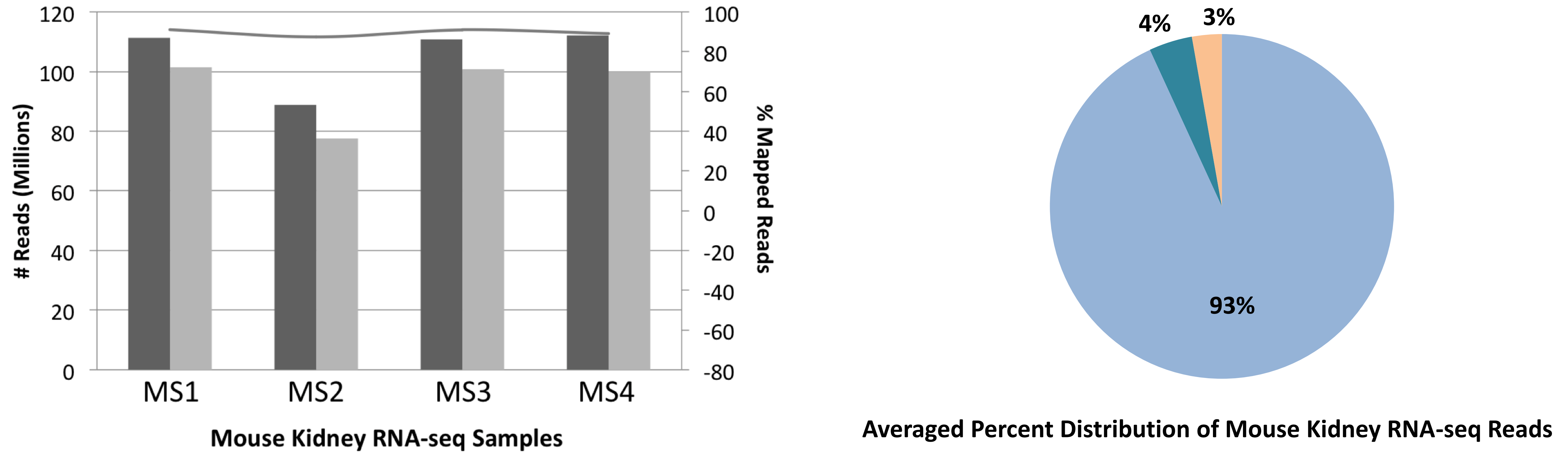

Reads (Millions)
% Mapped Reads
120
100
80
60
40
20
0
100
80
60
40
20
0
-20
-40
-60
-80
MS1
MS2
MS3
MS4
Mouse Kidney RNA-seq Samples
4%
3%
93%
Averaged Percent Distribution of Mouse Kidney RNA-seq Reads

S2A

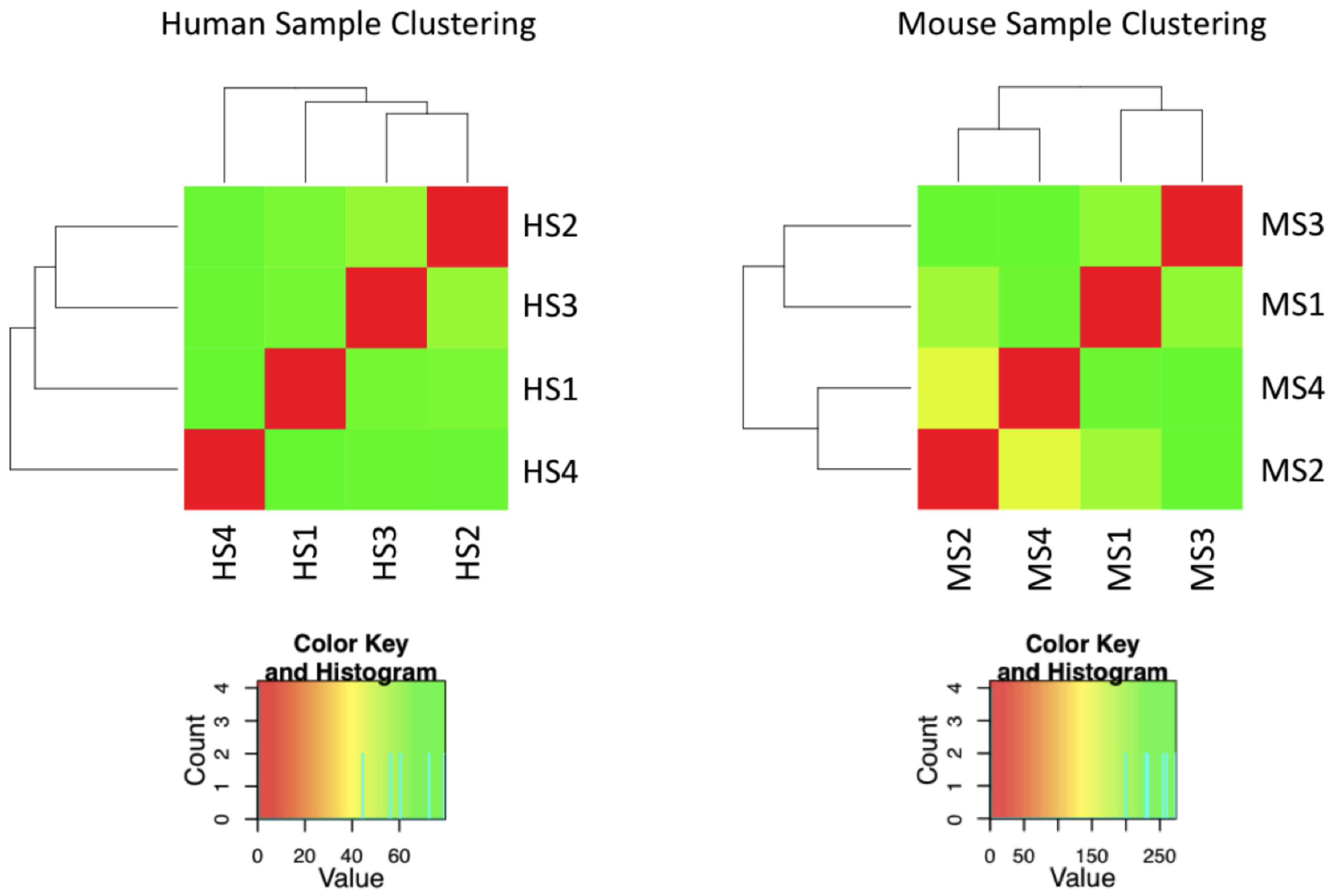
Human Sample Clustering
HS2
HS3
HS1
HS4
HS4
HS1
HS3
HS2
Color Key and Histogram
Count
0 1 2 3 4
0 20 40 60
Value
Mouse Sample Clustering
MS3
MS1
MS4
MS2
MS2
MS4
MS1
MS3
Color Key and Histogram
Count
0 1 2 3 4
0 50 150 250
Value

S2B

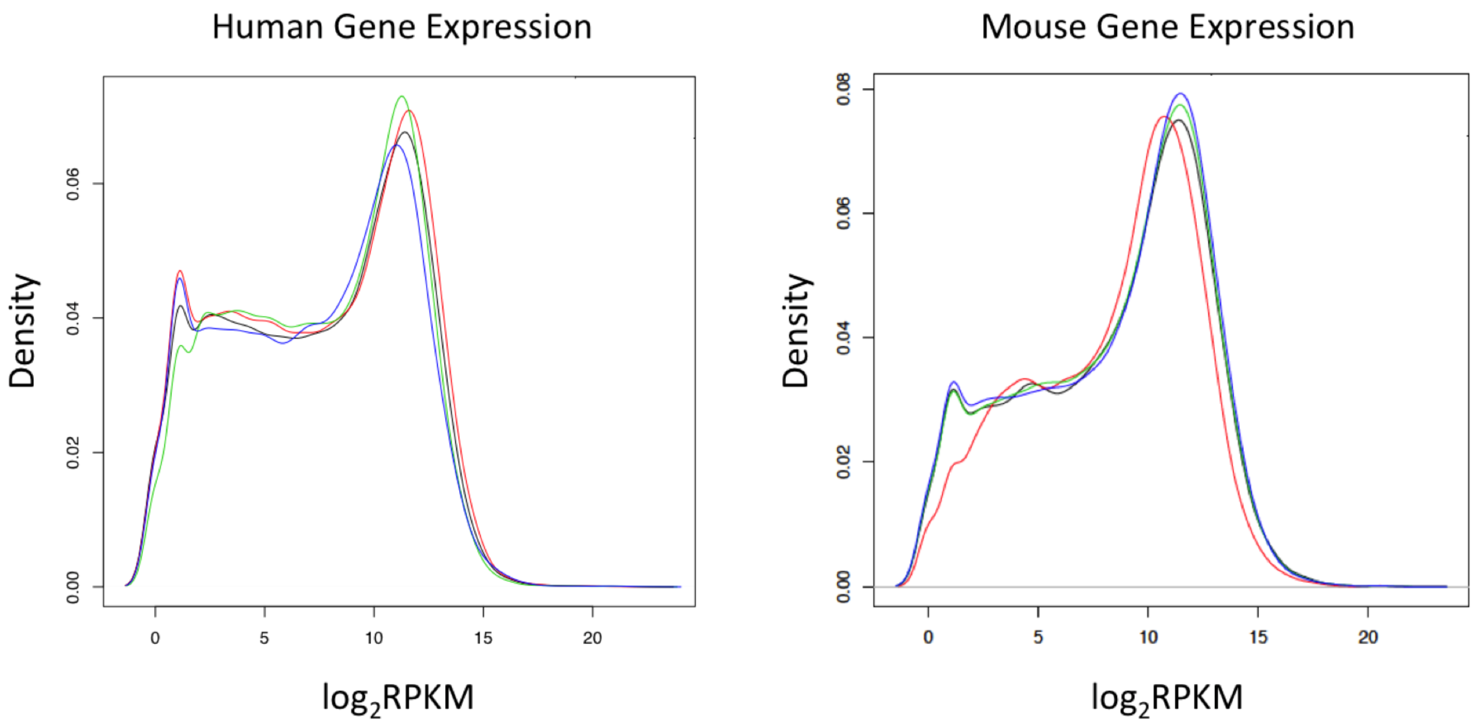
Human Gene Expression
Density
0.00 0.02 0.04 0.06
0 5 10 15 20
log_2RPKM
Mouse Gene Expression
Density
0.00 0.02 0.04 0.06 0.08
0 5 10 15 20
log_2RPKM

S3

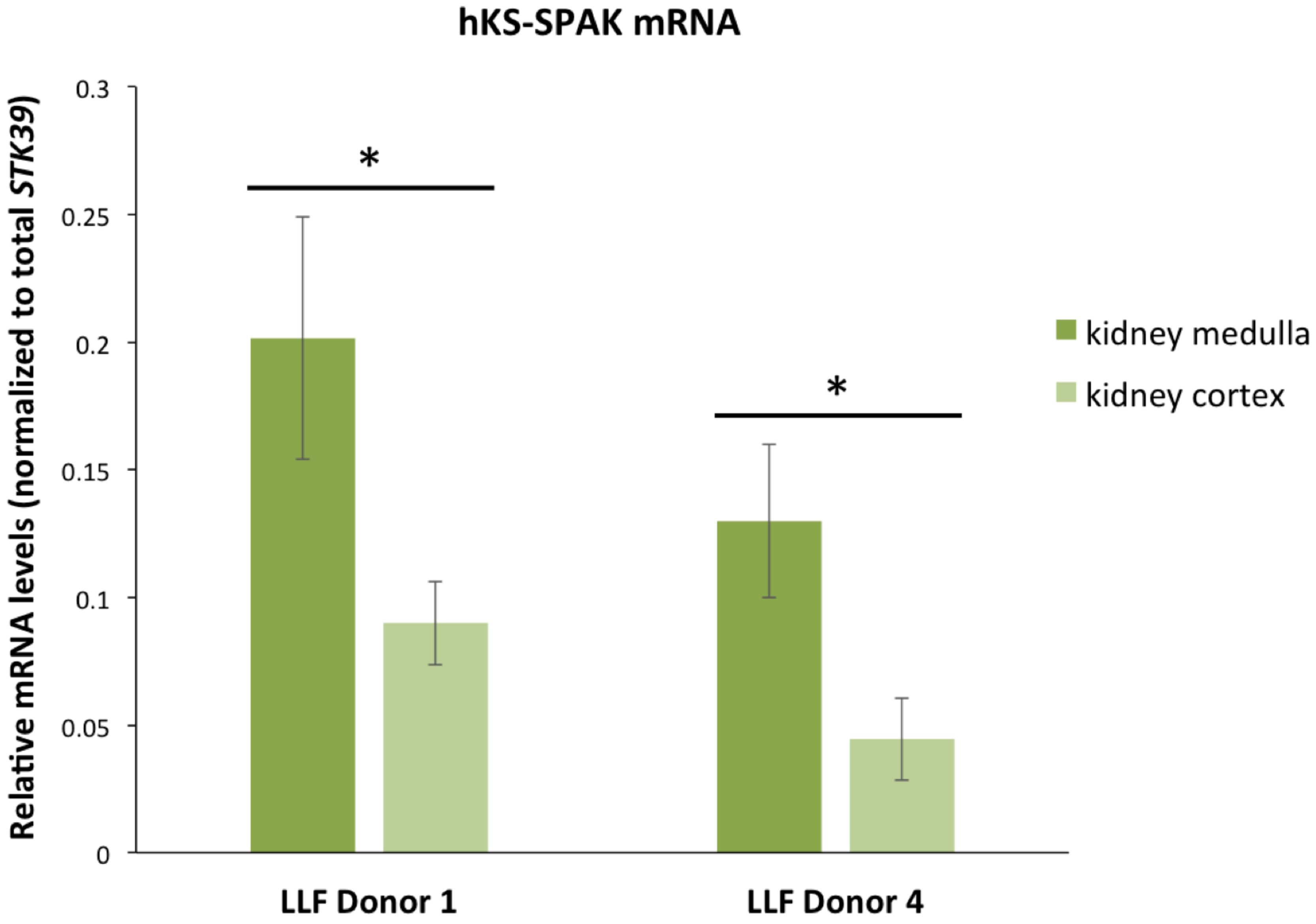
hKS-SPAK mRNA
Relative mRNA levels (normalized to total STK39)
0.3
0.25
0.2
0.15
0.1
0.05
0
*
*
LLF Donor 1
LLF Donor 4
kidney medulla
kidney cortex
